# Diagnostic Criteria for Depression in Type 2 Diabetes: A Data-Driven Approach

**DOI:** 10.1371/journal.pone.0112049

**Published:** 2014-11-12

**Authors:** Sergio E. Starkstein, Wendy A. Davis, Milan Dragovic, Violetta Cetrullo, Timothy M. E. Davis, David G. Bruce

**Affiliations:** 1 School of Medicine & Pharmacology, University of Western Australia, Crawley, Western Australia, Australia; 2 School of Psychiatry & Clinical Neuroscience, University of Western Australia, Crawley, Western Australia, Australia; Chiba University Center for Forensic Mental Health, Japan

## Abstract

**Background:**

While depression is a frequent psychiatric comorbid condition in diabetes and has significant clinical impact, the syndromal profile of depression and anxiety symptoms has not been examined in detail.

**Aims:**

To determine the syndromal pattern of the depression and anxiety spectrum in a large series of patients with type 2 diabetes, as determined using a data-driven approach based on latent class analysis (LCA).

**Method:**

Type 2 diabetes participants from the observational community-based Fremantle Diabetes Study Phase II underwent assessment of lifetime depression using the Brief Lifetime Depression Scale, the Patient Health Questionnaire 9-item version (PHQ-9) for current depression symptoms, and the Generalized Anxiety Disorder Scale that was specifically developed and validated for this study. The main outcome measure was classes of patients with a specific syndromal profile of depression and anxiety symptoms based on LCA.

**Results:**

LCA identified four classes that were interpreted as “major anxious depression”, “minor anxious depression”, “subclinical anxiety”, and “no anxious depression”. All nine DSM-IV/5 diagnostic criteria for major depression identified a class with a high frequency of major depression. All symptoms of anxiety had similar high probabilities as symptoms of depression for the “major depression-anxiety” class. There were significant differences between classes in terms of history of depression and anxiety, use of psychoactive medication, and diabetes-related variables.

**Conclusions:**

Patients with type 2 diabetes show specific profiles of depression and anxiety. Anxiety symptoms are an integral part of major depression in type 2 diabetes. The different classes identified here provide empirically validated phenotypes for future research.

## Introduction

Depression is one of the most frequent psychiatric disorders in patients with type 2 diabetes, with a lifetime prevalence of 24%–29% and a point prevalence of 10%-15% [1]. Depression in type 2 diabetes is significantly associated with poor glycaemic control [2], chronic complications [2], increased mortality [3], reduced physical and mental functioning [4], higher health costs and decreased adherence to diet and hypoglycaemic medications [5]. A recent epidemiological study demonstrated that individuals with diabetes and comorbid depression have a worse health-related quality of life and higher health service usage [6]. Collaborative care models for depression in diabetes demonstrated to significantly improve outcomes for depression and glycaemic control [7].

Depression may be underdiagnosed in diabetes. The limitations in diagnosing depression in diabetes include the lack of specific diagnostic criteria and the overlap of symptoms. Egede and Ellis [8] noted that poor management of diabetes is a barrier for early recognition of depressive symptoms, such as fatigue, changes in weight and appetite, sleep disturbances, and motor retardation. Furthermore, increased appetite is a cardinal diagnostic criterion for both major depression and diabetes, anhedonia may result from changes in lifestyle due to diabetes, loss of interest may be secondary to limitations to pursue usual free-time activities due to diabetes complications (e.g., visual problems, neuropathy), and loss of libido and reduced self-esteem may result from sexual dysfunction. Campayo and co-workers [9] suggested that depression in diabetic patients may be qualitatively different from depression observed among individuals without diabetes who have ‘primary’ (i.e. no organic cause) depression, but no supporting data were provided. A recent large prospective study showed that individuals with current depressive and/or anxiety disorders fad a 10-fold increased odds of diabetes incidence at two years, and the incidence was highest for those with comorbid depression and anxiety [10]. A valid and timely diagnosis of depression is of clinical relevance especially given that detection and treatment of depression improves glycaemic control [9].

Another potential confounder for a valid diagnosis of depression in diabetes is the potential comorbidity with anxiety. This is substantial in the general population, with 57% of individuals with major depression also meeting diagnostic criteria for an anxiety disorder [11]. Generalized anxiety disorder (GAD) is more frequent in diabetes than in age-comparable healthy controls [12], and is associated with poor health behaviours [13]. In the general population, comorbid depression and anxiety is highly prevalent [14]. Several studies examined the comorbidity between depression and anxiety in diabetes, but they were limited by important methodological shortcomings, such as a diagnosis of diabetes based only on self-report [13].

The aim of the present study was to examine the syndromal pattern of depression and anxiety in type 2 diabetes using a data-driven approach, as well as to examine the overlap with anxiety. We used baseline data from community-based participants of the Fremantle Diabetes Study Phase II (FDS2) [15], and used latent class analysis (LCA) to examine the homogeneity of depressive symptoms and the comorbidity between depression and anxiety. LCA assesses the symptom profile of individual patients and produces classes of patients in terms of their pattern of symptoms. Given that the classes thus identified do not overlap, individual patients can belong to only one group. Based on our experience with LCA in medical conditions, we expected to find three classes characterized by high, moderate and low probabilities for symptoms of depression, as well as a high comorbidity between depression and anxiety.

## Methods

### Patients

The FDS2 is a longitudinal observational study of known diabetes conducted in a postcode-defined geographical area surrounding the city of Fremantle (Western Australia) [15].

Details of FDS2 recruitment procedures, sample characteristics including classification of diabetes type and non-recruited patients have been published elsewhere [15]. Briefly, between 2008 and 2011, 4,667 diabetic patients were identified in the local population of 153,000 (crude diabetes prevalence 3%), of whom 1,668 (36%) were recruited to FDS2, in addition to 64 surviving FDS phase I participants who had moved out of the study area (1,732 participants altogether).

### Ethics Statement

The Human Research Ethics Committee of the Southern Metropolitan Area Health Service specifically approved the study, and all subjects gave written informed consent.

### Clinical and psychiatric assessment

Each participant underwent a baseline assessment that included a comprehensive clinical questionnaire, physical examination and fasting biochemical tests [15]. Diabetes type was assessed from treatment history, BMI, age at diagnosis, and nature of first presentation and/or self-identification. Non-insulin treated patients and those ≥60 years of age at diagnosis were usually considered to have type 2 diabetes, as were patients <60 years of age at diagnosis and taking insulin at the time of the study entry but whose first treatment was not insulin. In these cases, case records were consulted for evidence of ketonemia, as well as islet cell antibody, GAD antibodies, serum insulin and C-peptide levels, if available. Complications were ascertained using standard criteria [15], [16].

All participants were assessed with the PHQ-9 [17], a brief and accurate assessment of depression that has been validated in patients with diabetes [16]. The PHQ-9 is a dual-purpose instrument that can establish depressive disorder diagnoses as well as ratings of depressive symptom severity [17]. Patients with type 2 diabetes have significant physical complaints that may artificially increase PHQ-9 scores on items that are not necessary symptoms of major depression. Using a PHQ-9 cut point rather than the diagnostic algorithm may increase the frequency of false-positive cases of depression [18]. Therefore, as suggested by the PHQ-9 authors [18], we used the instrument's diagnostic algorithm for making diagnoses of major and minor depression. Major depression was diagnosed when the first or second symptoms (i.e. depressed mood or loss of interest/anhedonia) were present more than half the days, and at least 5 of the 9 symptoms were present during the same period [19]. Minor depression was diagnosed whenever 2 to 4 symptoms (including depressed mood or loss of interest/anhedonia) were present for more than half the days. Lifetime history of depression was assessed with the Brief Lifetime Depression Scale (BLDS). This instrument was developed and validated by our group for use in diabetes [20]. The BLDS was modelled on the PHQ-9 and included similar items, general format and language, but the participants were asked whether they had ever had a period during their lives lasting for two weeks or more when they experienced any of the nine symptoms of depression listed in the DSM-IV/5.

For the study of anxiety in the FDS2, a specific questionnaire (the Generalized Anxiety Disorder Scale (GADS)) was devised by one of the authors (SES) with experience in developing psychiatric instruments to rate the severity of anxiety (Table 1). The GADS includes 9 items that correspond to all 9 DSM-IV criteria for GAD. Based on the PHQ-9, GADS items were rated as “not at all present”, “present several days”, “present more than half of the days”, and “present nearly every day”. As with the PHQ-9, a symptom was rated positive whenever the patient rated “more than half of the days” or “nearly every day”. To make the instrument comparable to the DSM-IV criteria for GAD, the time criterion was the past 6 months. The validity of the GADS was examined by having a psychiatrist (SES) assess a convenience sample of 23 patients with the Structured Clinical Interview for the DSM-IV-Research Version (SCID)-Anxiety Disorders Module, blind to the GADS results. Kappa statistics showed a high diagnostic concordance for DSM-IV GAD diagnosis (kappa  = 0.88). Six of the 23 patients received a diagnosis of GAD based on the SCID, as compared to 4 based on the GADS (McNemar's test, *P* = 0.50). Test-retest reliability was assessed in 25 patients, who completed the GADS twice, with an interval of (mean ± SD) 9±6 days. The intra-class correlation (ICC) was high (ICC  = 0.88). The presence of cognitive deficits was based on the following algorithm (Bruce et al., 2008): patients scoring ≤26 on the MMSE were further assessed with the Clinical Dementia Rating (CDR) (Hughes et al., 1982) to establish the presence of dementia. This instrument generates ratings ranging from normal cognition (CDR 0) to severity levels of dementia (CDR 1–3) and an intermediate category defines very mild cognitive impairment or questionable dementia (CDR 0.5).

**Table 1:** Generalized Anxiety Disorder Scale.

Over the last 6 months, how often have you been bothered by any of the following problems?					
		Not at all	Several days	More than half the days	Nearly every day
1. worrying excessively or being anxious about several things?		0	1	2	3
2. difficulty controlling the worries or found the worries interfering with your ability to focus on what you were doing?		0	1	2	3
3. feeling restless, keyed up or on edge?		0	1	2	3
4. feeling tense?		0	1	2	3
5. feeling tired, weak or exhausted easily?		0	1	2	3
6. difficulty concentrating or finding your mind going blank?		0	1	2	3
7. feeling irritable?		0	1	2	3
8. difficulty sleeping (falling asleep, waking in the middle of the night, early morning wakening, or sleeping excessively)?		0	1	2	3
9. the symptoms of anxiety caused you significant distress or impaired your ability to function at work, socially, or in some other important way?		0	1	2	3
10. Were you taking any drugs or medicines just before the symptoms began?					
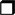 No	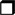 Yes	If YES, which drugs/medicines? _________________________			
_________________________________________________________________________					
11. 10b. Did you have any medical illness just before these symptoms began?					
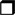 No	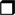 Yes	If YES, what was the illness? __________________________			
_________________________________________________________________________					

### Statistical Analysis

To test for group differences, generalised linear modelling (GLM) was used. LCA (cluster procedure) was used to determine the latent structure of patients with diabetes and to categorize individuals based on their responses to the PHQ-9 and GADS. LCA was calculated using the software LatentGold version 4.0.4 [21]. LCA assumes that a ‘class’ explains the association among a set of symptoms. LCA computes latent class probabilities or prevalence, and conditional probabilities. The basic assumption of LCA is that a (presumably homogeneous) population of individuals is a mixture of distinct, but internally homogeneous subgroups [22]. LCA assesses the symptom profile of individual patients and produces classes of patients as suggested by their pattern of symptoms. Since the clusters do not overlap, individual patients can belong to only one group. For this analysis, we included the nine DSM-IV symptoms for major depression and the four DSM-IV symptoms for generalized anxiety disorder that do not overlap with major depression as latent class indicators. Models with one to five classes were estimated. Each patient was assigned to the latent class to which the largest posterior probability was calculated. The best-fitting model was chosen based on having the lowest values for the following goodness of fit indices: Bayes Information Criterion (BIC), Approximate Weight of Evidence (AWE) (which takes into account classification performance), and the Corrected Akaike Information Criterion (CAIC). The level of contribution of each indicator to the final latent class structure is assessed by the information content statistic (*R^2^*), which can be interpreted similarly to the commonalities in traditional factor analysis. For the clinical interpretation, the mean level and frequency of responses for each psychiatric symptom within each class were examined. After determining the best class solution, we used latent class membership as a between-subject variable in a one-way analysis of variance (ANOVA) followed by post-hoc comparisons using Tukey's HSD, and *X^2^* analyses to examine demographic and clinical differences among the latent classes. The computer package IBM SPSS Statistics 20 (IBM Corporation, Somers, NY, USA) was used to compare baseline characteristics across the four LCA classes. Data are presented as proportions, mean ± SD, geometric mean (SD range), or, in the case of variables which did not conform to a normal or log-normal distribution, median [inter-quartile range, IQR]. For independent samples, two-way comparisons for proportions were by Fisher's exact test, for normally distributed variables by Student's *t*-test, and for non-normally distributed variables by Mann-Whitney U-test. Multiple comparisons for proportions were by Fisher's exact test or Chi-squared test, for normally distributed variables by one-way ANOVA, and for non-normally distributed variables by Kruskal-Wallis H-test, adjusted using the Bonferroni correction. A two-tailed significance level of *P*<0.05 was used throughout.

## Results

### Demographic and clinical findings

FDS2 recruited 1,551 participants (90%) with clinically defined type 2 diabetes. Complete data for our study was obtained from 1,337 patients (86%). When compared with the 214 patients with missing data, the latter were more likely to be female (46% vs. 57%, *P* = 0.005), and had worse glycaemic control (HbA_1c_ (median [IQR]) 6.8% median [IQR] [6.2%–7.6%] vs. 7.0% [6.4%–8.5%], respectively, *P* = 0.001). On the other hand, there were no significant between-group differences in age (mean years ± SD) (65.8±11.1 vs. 64.9±14.4 years, respectively, *P* = 0.41), diabetes duration (8.8 years [2.8–15.6] vs. 10.0 years [3.0–17.8], respectively, *P* = 0.12), or cognition (8.0% vs. 7.1% cognitively impaired (P = 1.00).

### Latent class analysis

**Figure 1:**
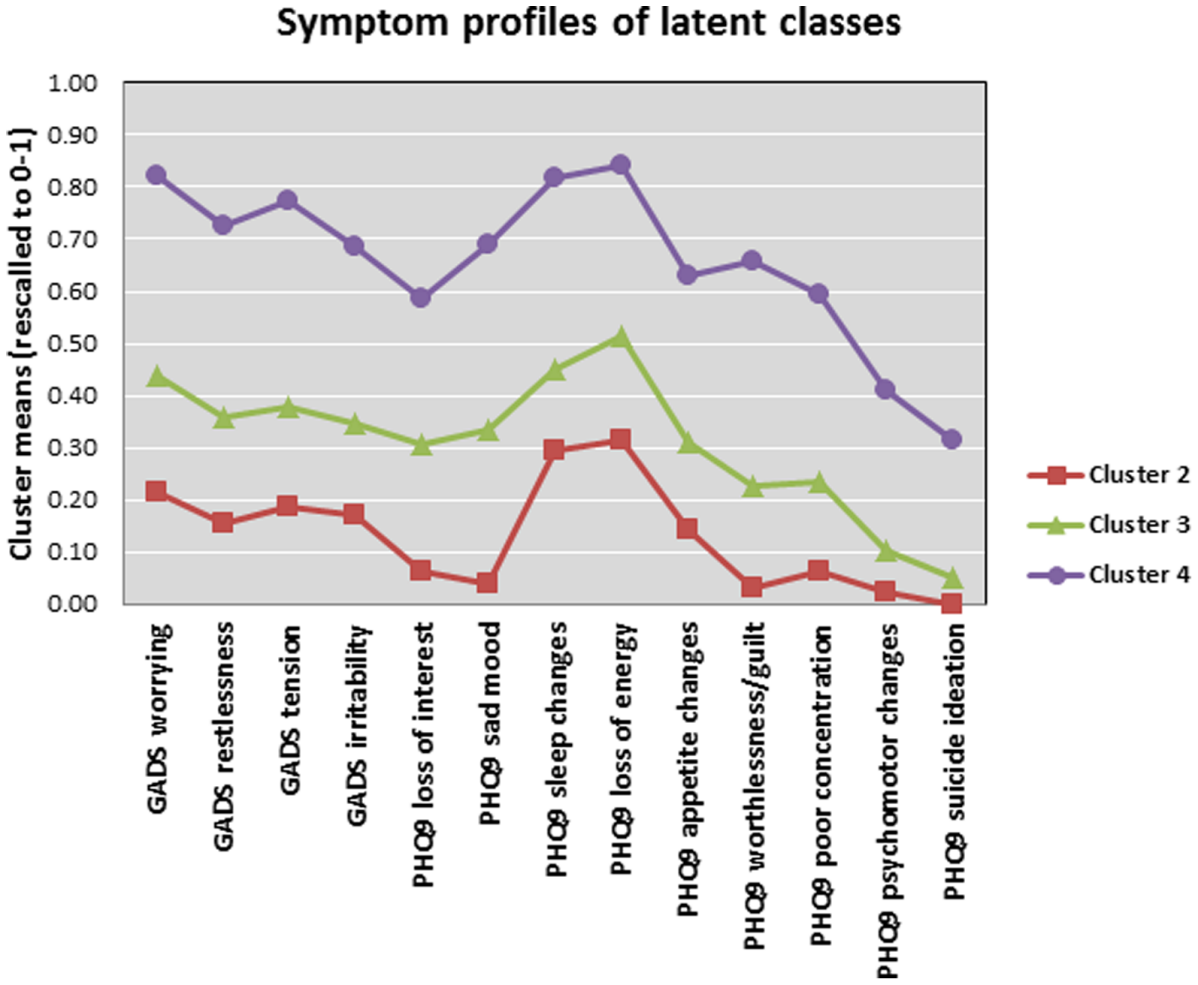
Symptom profile of the 4 (ordered) latent class model graph showing partial conditional probabilities for the 4-class model (LCA response profiles).

Items selected for the LCA included all 9 items from the PHQ-9, and the items of worrying/anxiety, feeling restless, feeling tense, and feeling irritable from the GADS (the remaining items were not included due to overlap with PHQ-9 items). The modelling started with probing the presence of conventional latent classes (i.e., cluster modelling), running cluster models of up to 5 latent clusters. Inspection of conditional probabilities showed a reduction in BIC and CAIC values from the 2- to the 5-cluster model, but at the cost of increasing number of parameters (NPar) (Table 2). From the 4-class model onwards, decreased accuracy was unacceptably high (i.e., >10%) and the AWE index has started to ascend. The 4-cluster model showed a pattern of probabilities extending from one extreme (low probabilities for all symptoms) to the other (high probabilities for all symptoms), with the exception of two intermediate classes showing a mixed pattern of probabilities. This finding suggests that a single discrete factor (anxious depression) with 4 ordered levels or classes (e.g., no anxious depression, subclinical anxiety, minor anxious depression and major anxious depression) provides the best explanatory model. Thus, an inter-factorial effect and orderliness of latent classes was obtained, as opposed to random and/or pseudonominal composition of latent classes. Class-specific endorsement profiles for depression and anxiety symptoms are shown in Figure 1.

**Table 2:** Testing Six Subsequent Latent Class Analysis Models.

Number of classes	*L* ^2^	Reduction from baseline	Gain (%)	Classification error	BIC*	AWE*	CAIC
**One class**	17470.9	-		0.00	33396.3	33797.7	33435.3
**Two classes**	12744.7	27.1	27.1	0.04	28772.2	29555.7	28825.3
**Three classes**	11449.9	34.5	7.4	0.06	27579.6	28733.8	27646.6
**Four classes**	10988.6	37.8	2.6	0.10	27222.3	28785.1	27301.2
**Five classes**	10720.2	38.6	1.5	0.13	27053.9	28964.8	27148.9

* Information criteria evaluating the quality of latent class solution: L2 - The likelihood-ratio goodness-of-fit, BIC (Bayes Information Criterion), AWE (Approximate Weight of Evidence, similar to BIC but also takes classification performance into account), CAIC (Corrected Akaike Information Criterion).

Latent class 4 included 104 patients (8%) and showed high partial conditional probabilities (>0.60) for all the anxiety and depression symptoms, except for psychomotor changes (>0.40) and suicidal ideation (>0.30). This class was labelled “major anxious depression”. Latent class 1 included 439 patients (33%) and showed low partial conditional probabilities (<0.10) for all symptoms of depression and anxiety. This class was labelled “no anxious depression”. Latent class 3 included 293 patients (22%) with partial conditional probabilities intermediate between classes 1 and 4, and was labelled “minor anxious depression”. Finally, latent class 2 included 501 patients (38%) and showed higher conditional probabilities than class 4 on the anxiety items, sleep changes and loss of energy. This class was labelled “subclinical anxiety”. We used final class membership as a between-subject variable to evaluate differences in depressive and anxiety symptoms as a function of latent class membership. The ANOVAs evaluating mean symptom severity differences on each item as a function of latent class membership were all significant at the *P*<0.001 level. Follow-up pairwise comparisons using Tukey's HSD tests demonstrated the following differences: For GAD worry the ANOVA was significant (Table 3), and all four groups were significantly different to each other (Tukey HSD *P* = 0.0001). Similar results were obtained for GADS restlessness, GADS tension, GADS irritability, loss of interest, sad mood, sleep changes, loss of energy, appetite changes, worthlessness/guilt, and poor concentration. For the items of psychomotor changes and suicide ideation there were significant differences on post-hoc tests between all classes except between classes 1 and 2.

**Table 3:** Scores for anxiety and depression items for the four LCA classes.

Symptom	No anxious –depression	Subsyndromal anxiety	Minor anxious depression	Major anxious depression	F	*P*
**Worrying**	0.06 (0.23)	0.65 (0.63)	1.33 (0.87)	2.49 (0.73)	548.6	0.0001
**Restlessness**	0.01 (0.09)	0.47 (0.53)	1.09 (0.70)	2.17 (0.83)	597.3	0.0001
**Tension**	0.01 (0.10)	0.57 (0.55)	1.15 (0.72)	2.33 (0.74)	654.6	0.0001
**Irritability**	0.06 (0.24)	0.52 (0.58)	1.04 (0.69)	2.06 (0.89)	430.5	0.0001
**Loss of interest**	0.02 (0.17)	0.18 (0.45)	0.96 (0.84)	1.75 (0.91)	398.3	0.0001
**Sad mood**	0.02 (0.13)	0.12 (0.33)	1.03 (0.74)	2.06 (0.81)	754.3	0.0001
**Sleep changes**	0.28 (0.66)	0.90 (1.00)	1.35 (1.06)	2.47 (0.85)	162.7	0.0001
**Loss of energy**	0.20 (0.43)	0.96 (0.83)	1.55 (0.97)	2.55 (0.68)	204.8	0.0001
**Appetite changes**	0.09 (0.33)	0.44 (0.78)	0.94 (1.03)	1.88 (1.17)	179.0	0.0001
**Worthlessness/guilt**	0.01 (0.48)	0.09 (0.30)	0.70 (0.77)	1.96 (1.08)	131.1	0.0001
**Poor concentration**	0.01 (0.08)	0.19 (0.47)	0.72 (0.87)	1.76 (1.04)	104.0	0.0001
**Psychomotor changes**	0.01 (0.04)	0.08 (0.34)	0.30 (0.59)	1.25 (1.17)	207.7	0.0001
**Suicide ideation**	0.00 (0.00)	0.01 (0.04)	0.17 (0.50)	0.91 (1.09)	57.7	0.0001

There were significant demographic and clinical differences between classes (Table 4). The *major anxious depression class* was significantly younger than the other classes, whilst the *minor anxious depression class* was significantly younger than the subclinical anxiety class. Depression (major or minor) was significantly more frequent in the *major anxious depression class* as compared to the other classes, and in the *minor anxious depression class* as compared to the remaining two classes. Depression before the onset of diabetes was significantly higher in the *major anxious depression class* as compared to the other classes, and in the *minor anxious depression class* as compared to the remaining two classes. Patients in the *major anxious depression class* had a significantly higher frequency of GAD as compared to the other classes, whilst patients in the *minor anxious depression class* had a significantly higher frequency of GAD than the remaining two classes. Depression (minor or major) and/or GAD were present in 89% of patients in the *major anxious depression class*, 29% in the *minor anxious depression class*, 2% in the *subclinical anxiety class*, and none in the *no anxious depression class*.

**Table 4:** FDS2 baseline associates of four LCA groups in participants with type-2 diabetes.

	LCA group				
FDS2 baseline variable	No anxious depression (Cluster 1)	Subsyndromal anxiety (Cluster 2)	Minor anxious depression (Cluster 3)	Major anxious depression (Cluster 4)	*P*-value
**N (%)**	439 (32.8)	501 (37.5)	293 (21.9)	104 (7.8)	
**Age (years)**	67.4±10.8	66.1±10.8	64.7±11.1††	60.6±12.3*** ^,^ ††† ^,^ ‡‡	**<0.001**
**Any depressive syndrome (%)**	0	1.0	26.3*** ^,^ †††	77.9*** ^,^ ††† ^,^ ‡‡‡	**<0.001**
**Major depressive syndrome (%)**	0	0	4.1*** ^,^ †††	67.3*** ^,^ ††† ^,^ ‡‡‡	**<0.001**
**Taking antidepressant medication (%)**	6.6	13.6**	23.5*** ^,^ †††	35.6*** ^,^ †††	**<0.001**
**No depression/depression before diabetes/concomitant diagnoses/depression after diabetes (%)**	88.2/7.7/1.2/3.0	64.0/19.6/5.4/11.0***	29.8/32.4/10.2/27.6*** ^,^ †††	2.1/43.3/16.5/38.1*** ^,^ ††† ^,^ ‡‡‡	**<0.001**
**Current GAD (%)**	0	0	3.8*** ^,^ †††	51.0*** ^,^ ††† ^,^ ‡‡‡	**<0.001**
**Lifetime GAD (%):**	5.1	14.7***	35.6*** ^,^ †††	70.3*** ^,^ ††† ^,^ ‡‡‡	**<0.001**
**Any depressive syndrome and/or current GAD (DSMIV) (%)**	0	1.6	29.4*** ^,^ †††	89.4*** ^,^ ††† ^,^ ‡‡‡	**<0.001**
**Male (%)**	58.5	52.3	50.2	45.2	**0.030**
**Educated beyond primary level (%)**	88.2	88.1	88.5	80.4	0.17
**Currently married**	65.1	65.9	63.1	63.5	0.87
**Alcohol consumption (standard drinks/day)**	0.1 [0–1.5]	0.1 [0–1.2]	0.1 [0–1.5]	0 [0–0.3]*** ^,^ † ^,^ ‡‡	**0.003**
**Age at diagnosis of diabetes (years)**	57.4±11.7	56.2±11.5	54.7±11.5†	49.9±11.6*** ^,^ ††† ^,^ ‡‡	**<0.001**
**Diabetes duration (years)**	8.5 [3.0–15.8]	8.0 [2.3–15.4]	9.0 [2.0–15.5]	10.0 [4.0–15.5]	0.57
**Fasting serum glucose (mmol/L)**	7.1 [6.2–8.7]	7.1 [6.2–8.5]	7.1 [6.1–8.9]	8.1 [6.2–10.3]* ^,^ †	**0.021**
**HbA_1c_ (%)**	6.8 [6.3–7.5]	6.7 [6.2–7.5]	6.8 [6.2–7.6]	7.5 [6.3–8.9]*** ^,^ ††† ^,^ ‡‡‡	**0.001**
**Diabetes treatment (% diet/OGLMs/insulin/insulin + OGLMs)**	23.9/57.2/3.2/15.7	27.1/52.3/3.4/17.2	24.9/51.2/6.8/17.1	18.3/43.3/9.6/28.8*** ^,^ ††	**0.001**
**BMI (kg/m^2^)**	30.9±5.9	30.9±5.8	32.0±6.6	33.5±7.0** ^,^ ††	**<0.001**
**Central adiposity (% obese by waist circumference)**	68.6	70.9	74.4	83.5* ^,^ †	**0.013**
**Total serum cholesterol (mmol/L)**	4.3±1.0	4.4±1.0	4.4±1.2	4.5±1.3	0.09
**HDL-cholesterol (mmol/L)**	1.26±0.35	1.24±0.34	1.24±0.34	1.15±0.27	**0.033**
**Serum triglycerides (mmol/L)**	1.4 (0.9–2.4)	1.5 (0.9–2.5)	1.5 (0.9–2.5)	2.0 (1.0–3.8)*** ^,^ ††† ^,^ ‡‡‡	**<0.001**
**eGFR <60 ml/min/1.73 m^2^ (%)**	16.5	18.8	18.2	22.3	0.52
**Peripheral sensory neuropathy (%)**	57.6	59.7	61.6	56.3	0.67
**Any retinopathy (%)**	20.6	21.9	22.9	24.5	0.83
**Self-reported MI/angina (%)**	15.5	14.8	22.5†	25.0	**0.005**
**Self-reported stroke (%)**	2.5	4.0	6.5	7.7	**0.018**
**Peripheral arterial disease (%)**	23.5	20.2	21.8	24.3	0.59

*P<0.05,

**P<0.01,

***P<0.001 vs no depression-anxiety;

† *P*<0.05,

†† *P*<0.01,

††† *P*<0.001 vs minor depression-anxiety;

‡ *P*<0.05,

‡‡ *P*<0.01,

‡‡‡ *P*<0.001 vs subsyndromal anxiety after Bonferroni adjustment for multiple comparisons.

Finally, the *major anxious depression class* showed a significantly younger age at onset of diabetes, higher HbA_1c_ levels, higher serum triglycerides levels and a higher frequency of insulin use than the other three classes. This class also showed a higher BMI than the *subclinical anxiety* and the *no anxious depression classes*. When the class with major anxious-depression was compared with patients meeting DSM-IV criteria for major depression, there were no differences on demographic, diabetes-related or psychiatric variables (Table 5).

**Table 5:** FDS2 baseline associates of major anxious depression class vs major depression (DSM-IV criteria) in participants with type-2 diabetes.

FDS2 baseline variable	Major depression (DSM-IV)	Major anxious-depression (Cluster 4)
**N (%)**	82 (6.1)	104 (7.8)
**Age (years)**	60.6±12.5	60.6±12.3
**Any depressive syndrome (%)**	100.0	77.9
**Major depressive syndrome (%)**	100.0	67.3
**Taking antidepressant medication (%)**	42.7	35.6
**No depression/depression before diabetes/concomitant diagnoses/depression after diabetes (%)**	0/38.2/19.7/42.1	2.1/43.3/16.5/38.1
**Current GAD (%)**	43.9	51.0
**Lifetime GAD (%):**	67.1	70.3
**Any depressive syndrome and/or current GAD (DSMIV) (%)**	100.0	89.4
**Male (%)**	46.3	45.2
**Educated beyond primary level (%)**	79.0	80.4
**Currently married**	59.8	63.5
**Alcohol consumption (standard drinks/day)**	0 [0–0.4]	0 [0–0.3]
**Age at diagnosis of diabetes (years)**	49.3±11.3	49.9±11.6
**Diabetes duration (years)**	11.0 [4.0–16.1]	10.0 [4.0–15.5]
**HbA_1c_ (%)**	7.5 [6.2–9.0]	7.5 [6.3–8.9]
**Diabetes treatment (% diet/OGLMs/insulin/insulin + OGLMs)**	22.0/41.5/9.8/26.8	18.3/43.3/9.6/28.8
**BMI (kg/m^2^)**	33.5±6.1	33.5±7.0
**Central adiposity (% obese by waist circumference)**	85.4	83.5
**Total serum cholesterol (mmol/L)**	4.5±1.4	4.5±1.3
**HDL-cholesterol (mmol/L)**	1.14±0.29	1.15±0.27
**Serum triglycerides (mmol/L)**	2.0 (1.0–3.8)	2.0 (1.0–3.8)
**eGFR <60 ml/min/1.73 m^2^ (%)**	18.5	22.3
**Peripheral sensory neuropathy (%)**	53.7	56.3
**Any retinopathy (%)**	21.8	24.5
**History of ischaemic heart disease (%)**	36.6	25.0
**History of cerebrovascular disease (%)**	12.2	7.7
**Peripheral arterial disease (%)**	23.2	24.3

## Discussion

To our knowledge, this is the first study to examine the validity of diagnostic criteria for depression in diabetes using LCA in a large community sample of patients with type 2 diabetes, and there were several important findings. First, LCA of depressive and anxiety symptoms from our sample produced evidence for a 4-class solution: a *major depression-anxiety class*, a *minor depression-anxiety class*, a *subsyndromal anxiety class*, and a class with *no depression or anxiety*. Second, all nine DSM-IV diagnostic criteria for major depression identified a class of patients with a high frequency of major depression, and further changes to these criteria are not necessary. Third, all symptoms of anxiety had similar high probabilities as symptoms of depression for the *major depression-anxiety class*, suggesting that anxiety symptoms should be added to the DSM-IV/5 diagnostic criteria for major depression to identify a class with more severe psychopathology. Therefore, our findings do not support separating anxiety from depressive symptoms for patients with type 2 diabetes. Fourth, the *major depression-anxiety class* had worse diabetes-related parameters than the class without anxiety or depression, suggesting that anxious depression is a marker of more malignant diabetes. Finally, we also identified a class that included anxiety and depression symptoms of a lesser severity, which we termed *minor depression-anxiety*. About one third of this group had clinically relevant anxiety and/or depression, but there were no demographic or clinical differences when compared to the class without anxiety or depression.

These findings should be considered in the light of some limitations of our study. First, given the large sample of individuals to be assessed and the comprehensive quantity of clinical assessments, we were unable to interview patients with more appropriate structured psychiatric interviews. Nevertheless, we assessed depression using the PHQ-9, a valid and reliable instrument to diagnose depression based on DSM-IV criteria and to measure the severity of depression. Second, anxiety symptoms were assessed with the GADS, a new instrument specifically developed and validated for the present study. As with the PHQ-9, the GADS allows a diagnosis of GAD based on DSM-IV criteria and measures the severity of anxiety. We did not use the Generalized Anxiety Disorder 7-item scale (GAD-7) given that this instrument was not available when our study was designed. However, we believe that the GADS is an improvement over the GAD-7 given that the GADS assesses all the DSM-IV/5 criteria for GAD over the past 6 months, whilst the GAD-7 assesses a partial list of symptoms during the past 2 weeks. This was a cross-sectional study, and the clinical progression of the classes identified in this study will need to be examined in our ongoing biennial follow-up study. Finally, we did not assess for the category of ‘diabetes distress’ in our study. The syndrome of major depression was criticized for not differentiating an expected emotional reaction to a significant stressor from a pathological reaction, whereas ‘diabetes distress’ may better capture the worries and fears of a chronic illness [23]. However, the construct of ‘diabetes distress’ is in need of further validation, and it is not included into the major psychiatric nomenclatures. Our study provides empirical support for a class that includes the symptoms of major depression in diabetes, and demonstrates this class to be significantly related to worse diabetes outcomes.

While depression is recognized as one of the most frequent psychiatric disorders in type 2 diabetes, questions remain regarding the validity of using DSM-IV/5 diagnostic criteria when the symptoms of depression and diabetes can overlap. Given the impact of depression on the management and prognosis of diabetes, it is critical to validate diagnostic criteria for depression for use in clinical practice. Using LCA, we found for the first time that all nine- DSM-IV criteria for major depression identify a class of patients with a high chance of having major depression. Moreover, this group also showed significantly worse clinical parameters, such as higher fasting plasma glucose, higher HbA1c, greater use of insulin, higher BMI, increased central adiposity and increased serum triglycerides than the class without anxious depression, which provides partial validation of the classes obtained.

The classes with high probability of depressive symptoms also included a high probability of anxiety symptoms (worrying, restlessness, tension and irritability) and had a high frequency of GAD. This finding demonstrates the high comorbidity of depression and anxiety in diabetes, and suggests that a new diagnostic category of “major anxious depression” should be used in type 2 diabetes. In the general population, this comorbidity is associated with a poor prognosis, recurrent depression, worse treatment outcomes of depression, more frequent suicide attempts, greater symptom severity, diminished social supports, and more past-year distressing life events [24]. Starr and Davila [25] proposed a model of depression-anxiety comorbidity in which anxiety symptoms lead to depressive symptoms via maladaptive anxiety response styles.

Another relevant finding from our study was a class with intermediate symptoms of anxiety and depression, which we labelled *minor depression-anxiety*. This class included a relatively large proportion of our sample (22%), and future studies may determine whether this class may evolve into a more severe depression with significant impact upon diabetes variables. Cyranowsky et al [24] recently demonstrated that anxiety commonly emerges alongside subthreshold depressive symptoms. The finding that 29% of this class had depression and/or anxiety suggests that this class is clinically relevant. It has been demonstrated that subclinical depression has a significant negative impact on diabetes control [26] and the relationship between depressive symptoms and poor self-care appears to be linear [1]. A third class, which included 38% of the sample, had minor symptoms of anxiety but no depression. Only 2% of this group had clinical depression and/or anxiety, but had a significantly higher lifetime prevalence of GAD and depression as compared to the class with no anxiety or depression. The clinical relevance of this class should be further examined in longitudinal studies.

In conclusion, we demonstrated that the DSM-IV criteria for major depression may be used unmodified to diagnose depression in diabetes. We also found a high comorbidity between depression and anxiety, suggesting that symptoms of anxiety may be included into a new set of diagnostic criteria for major depression or mixed anxiety-depression in type 2 diabetes. Future longitudinal studies will validate this construct and examine the clinical relevance of minor anxious depression and subclinical anxiety in type 2 diabetes.

## References

[1] Blay SL, Fillenbaum GG, Marinho V, Andreoli SB, Gastal FL (2011) Increased health burden associated with comorbid depression in older Brazilians with diabetes. J Affect Disord 134(1–3): 77–84. 21684613 10.1016/j.jad.2011.05.012 PMC3659776

[2] Gonzalez JS, Peyrot M, McCarl LA, Collins EM, Serpa L, et al (2008) Depression and diabetes treatment nonadherence: a meta-analysis. Diabetes Care 31(12): 2398–403. 19033420 10.2337/dc08-1341 PMC2584202

[3] de Groot M, Anderson R, Freedland KE, Clouse RE, Lustman PJ (2001) Association of depression and diabetes complications: a meta-analysis. Psychosom Med 63(4): 619–30. 11485116 10.1097/00006842-200107000-00015

[4] Bruce DG, Davis WA, Starkstein SE, Davis TM (2005) A prospective study of depression and mortality in patients with type 2 diabetes: the Fremantle Diabetes Study. Diabetologia 48(12): 2532–9. 16292463 10.1007/s00125-005-0024-3

[5] Markowitz SM, Gonzalez JS, Wilkinson JL, Safren SA (2011) A review of treating depression in diabetes: emerging findings. Psychosomatics 1 52(1): 1–18. 10.1016/j.psym.2010.11.007 PMC3043600 21300190

[6] Atlantis E, Goldney RD, Eckert KA, Taylor AW, Phillips P (2012) Trends in health-related quality of life and health service use associated with comorbid diabetes and major depression in South Australia, 1998–2008. Social psychiatry and psychiatric epidemiology 47(6): 871–7. 21590369 10.1007/s00127-011-0394-4

[7] Atlantis E, Fahey P, Foster J (2014) Collaborative care for comorbid depression and diabetes: a systematic review and meta-analysis. BMJ open 4(4): e004706. 10.1136/bmjopen-2013-004706 PMC3987739 24727428

[8] Egede LE, Ellis C (2010) Diabetes and depression: global perspectives. Diabetes research and clinical practice 87(3): 302–12. 20181405 10.1016/j.diabres.2010.01.024

[9] Campayo A, Gomez-Biel CH, Lobo A (2011) Diabetes and depression. Current psychiatry reports 13(1): 26–30. 21052874 10.1007/s11920-010-0165-z

[10] Atlantis E, Vogelzangs N, Cashman K, Penninx BJ (2012) Common mental disorders associated with 2-year diabetes incidence: the Netherlands Study of Depression and Anxiety (NESDA). J Affect Disord 142 Suppl: S30–5. 23062854 10.1016/S0165-0327(12)70006-X

[11] Kessler RC, Merikangas KR, Wang PS (2007) Prevalence, comorbidity, and service utilization for mood disorders in the United States at the beginning of the twenty-first century. Annual review of clinical psychology 3: 137–58. 10.1146/annurev.clinpsy.3.022806.091444 17716051

[12] Collins MM, Corcoran P, Perry IJ (2009) Anxiety and depression symptoms in patients with diabetes. Diabet Med 26(2): 153–61. 19236618 10.1111/j.1464-5491.2008.02648.x

[13] Edwards LE, Mezuk B (2012) Anxiety and risk of type 2 diabetes: evidence from the Baltimore Epidemiologic Catchment Area Study. J Psychosom Res 73(6): 418–23. 23148808 10.1016/j.jpsychores.2012.09.018 PMC3499773

[14] Nutt D, Argyropoulos S, Hood S, Potokar J (2006) Generalized anxiety disorder: A comorbid disease. European neuropsychopharmacology: the journal of the European College of Neuropsychopharmacology 16 Suppl 2: S109–18. 16737802 10.1016/j.euroneuro.2006.04.003

[15] Davis TM, Bruce DG, Davis WA (2013) Cohort profile: the Fremantle Diabetes Study. Int J Epidemiol 42(2): 412–21. 22544845 10.1093/ije/dys065

[16] Roy T, Lloyd CE, Pouwer F, Holt RI, Sartorius N (2012) Screening tools used for measuring depression among people with Type 1 and Type 2 diabetes: a systematic review. Diabet Med 29(2): 164–75. 21824180 10.1111/j.1464-5491.2011.03401.x

[17] Kroenke K, Spitzer RL, Williams JB (2001) The PHQ-9: validity of a brief depression severity measure. J Gen Intern Med 16(9): 606–13. 11556941 10.1046/j.1525-1497.2001.016009606.x PMC1495268

[18] Kroenke K, Strine TW, Spitzer RL, Williams JB, Berry JT (2009) The PHQ-8 as a measure of current depression in the general population. J Affect Disord 114(1–3): 163–73. 18752852 10.1016/j.jad.2008.06.026

[19] Kroenke K, Spitzer RL, Williams JB, Lowe B (2010) The Patient Health Questionnaire Somatic, Anxiety, and Depressive Symptom Scales: a systematic review. Gen Hosp Psychiatry 32(4): 345–59. 20633738 10.1016/j.genhosppsych.2010.03.006

[20] Bruce DG, Davis WA, Cetrullo V, Starkstein SE, Davis TM (2013) Clinical Impact of the Temporal Relationship between Depression and Type 2 Diabetes: The Fremantle Diabetes Study Phase II. PLoS One 8(12): e81254. 24324682 10.1371/journal.pone.0081254 PMC3852722

[21] Vermunt JK, Magidson J (2005) Technical Guide for Latent GOLD Choice 4.0: Basic and Advanced. Belmont, Massachussetts.

[22] Ruscio J, Brown TA, Meron Ruscio A (2009) A taxometric investigation of DSM-IV major depression in a large outpatient sample: interpretable structural results depend on the mode of assessment. Assessment 16(2): 127–44. 19234296 10.1177/1073191108330065 PMC3536496

[23] Fisher L, Gonzalez JS, Polonsky WH (2014) The confusing tale of depression and distress in patients with diabetes: a call for greater clarity and precision. Diabet Med 31(7): 764–72. 24606397 10.1111/dme.12428 PMC4065190

[24] Cyranowski JM, Schott LL, Kravitz HM, Brown C, Thurston RC, et al (2012) Psychosocial features associated with lifetime comorbidity of major depression and anxiety disorders among a community sample of mid-life women: the SWAN mental health study. Depress Anxiety 29(12): 1050–7. 22930404 10.1002/da.21990 PMC3592574

[25] Starr LR, Davila J (2012) Responding to Anxiety with Rumination and Hopelessness: Mechanism of Anxiety-Depression Symptom Co-Occurrence? Cognitive therapy and research 36(4): 321–37. 22865943 10.1007/s10608-011-9363-1 PMC3409687

[26] Fisher L, Skaff MM, Mullan JT, Arean P, Mohr D, et al (2007) Clinical depression versus distress among patients with type 2 diabetes: not just a question of semantics. Diabetes Care 30(3): 542–8. 17327318 10.2337/dc06-1614

