# Correction: Diagnostic Criteria for Depression in Type 2 Diabetes: A Data-Driven Approach

**DOI:** 10.1371/journal.pone.0122324

**Published:** 2015-03-23

**Authors:** The PLOS ONE Staff

In Fig. 1, the line and data points for Cluster 1 are missing. Please see a complete, correct version of Fig. 1 below.

**Fig 1:**
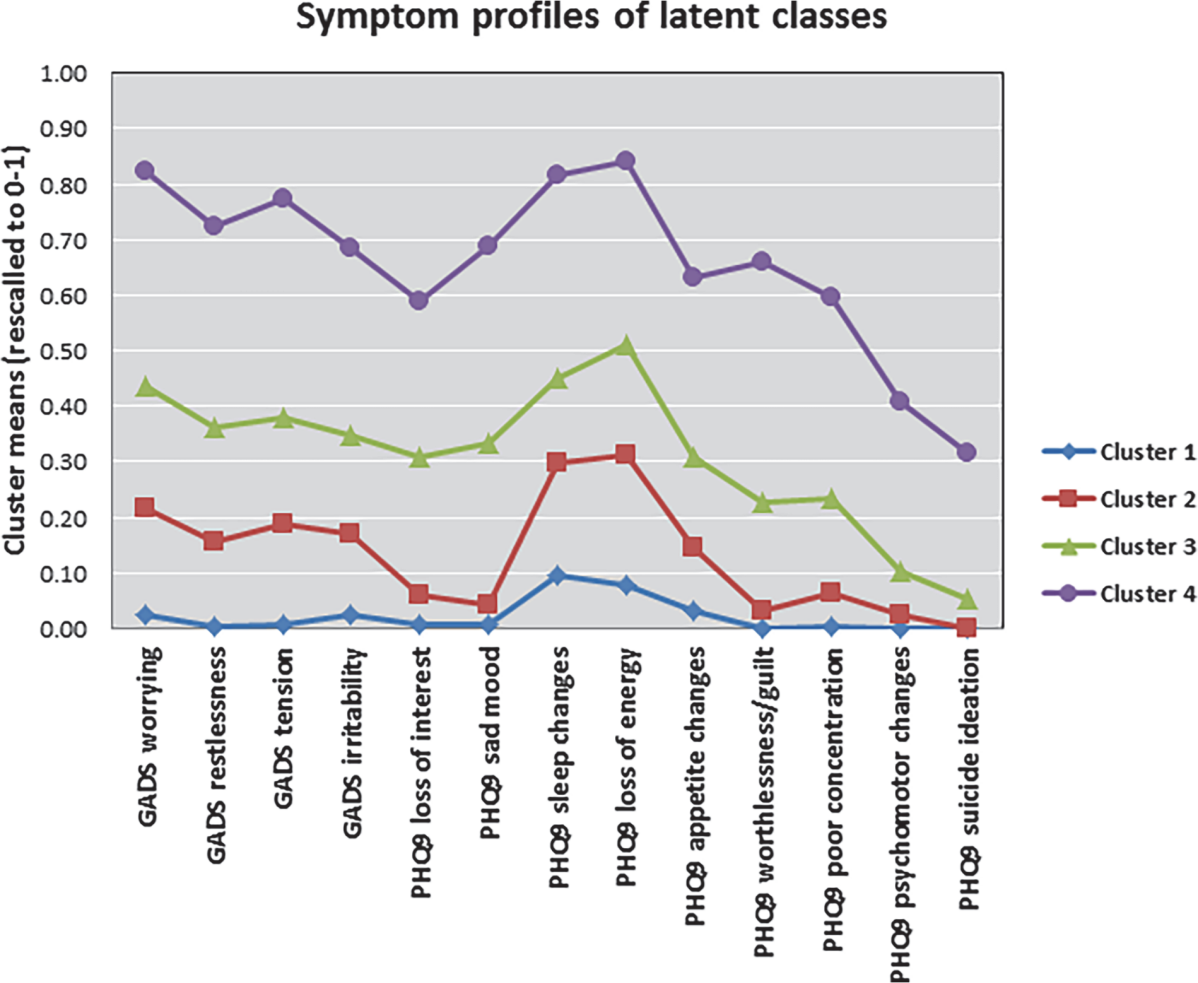
Symptom profile of the 4 (ordered) latent class model graph showing partial conditional probabilities for the 4-class model (LCA response profiles).
